# A robust mass spectrometry method for rapid profiling of erythrocyte ghost membrane proteomes

**DOI:** 10.1186/s12014-018-9190-4

**Published:** 2018-03-21

**Authors:** Haddy K. S. Fye, Paul Mrosso, Lesley Bruce, Marie-Laëtitia Thézénas, Simon Davis, Roman Fischer, Gration L. Rwegasira, Julie Makani, Benedikt M. Kessler

**Affiliations:** 1 Target Discovery Institute, Nuffield Department of Medicine, University of Oxford, Roosevelt Drive, Oxford, OX3 7FZ UK; 2 Muhimbili Wellcome Programme, Muhimbili University of Health and Allied Sciences, PO Box 65001, Dar es Salaam, Tanzania; 3 Bristol Institute for Transfusion Sciences, NHS Blood and Transplant, 500 North Bristol Park, Filton, Bristol, BS34 7QH UK

**Keywords:** Erythrocyte ghosts, Shotgun proteomics, Tandem mass spectrometry, Membrane proteome, Haemoglobinopathies

## Abstract

**Background:**

Red blood cell (RBC) physiology is directly linked to many human disorders associated with low tissue oxygen levels or anemia including chronic obstructive pulmonary disease, congenital heart disease, sleep apnea and sickle cell anemia. Parasites such as *Plasmodium* spp. and *phylum Apicomplexa* directly target RBCs, and surface molecules within the RBC membrane are critical for pathogen interactions. Proteomics of RBC membrane ‘ghost’ fractions has therefore been of considerable interest, but protocols described to date are either suboptimal or too extensive to be applicable to a larger set of clinical cohorts.

**Methods:**

Here, we describe an optimised erythrocyte isolation protocol from blood, tested for various storage conditions and explored using different fractionation conditions for isolating ghost RBC membranes. Liquid chromatography mass spectrometry (LC–MS) analysis on a Q-Exactive Orbitrap instrument was used to profile proteins isolated from the comparative conditions. Data analysis was run on the MASCOT and MaxQuant platforms to assess their scope and diversity.

**Results:**

The results obtained demonstrate a robust method for membrane enrichment enabling consistent MS based characterisation of > 900 RBC membrane proteins in single LC–MS/MS analyses. Non-detergent based membrane solubilisation methods using the tissue and supernatant fractions of isolated ghost membranes are shown to offer effective haemoglobin removal as well as diverse recovery including erythrocyte membrane proteins of high and low abundance.

**Conclusions:**

The methods described in this manuscript propose a medium to high throughput framework for membrane proteome profiling by LC–MS of potential applicability to larger clinical cohorts in a variety of disease contexts.

**Electronic supplementary material:**

The online version of this article (10.1186/s12014-018-9190-4) contains supplementary material, which is available to authorized users.

## Background

Whereas erythrocyte proteins, in particular globins, have been studied extensively, the application of proteomics methods to erythrocyte membrane ‘ghost’ preparations is a relatively recent phenomenon with the first experiments combining gel separation with Matrix Associated Laser Desorption Ionization Time-of-Flight (MALDI-TOF) analysis published in 2002 [1]. Since then, a growing number of original research papers have highlighted the benefits of RBC proteomics, in particular the membrane fraction, as well as drawing light to some of the challenges faced in its implementation and practice. In particular, the difficulties faced in obtaining pure fractions of RBC’s from whole blood, reducing cytosolic haemoglobin contamination as well as overcoming the wide dynamic range in protein expression. To facilitate the collection of pure sample fractions; a large number of methodologies utilizing centrifugation [2], filtration [3, 4], columns [5], flow cytometry [6], multiple enzyme digests combined with fractionation [7] as well as in vitro whole blood incubation to enable reticulocytes to mature over 72–96 h have been presented [3].

One of the more comprehensive published protocols proposes a combination of some of these approaches along with the use of a haemoglobin depletion kit to allow the collection of an enriched RBC fraction with minimal cytosolic contamination [8]. In addition to the diversity of erythrocyte isolation and purification methods, there exist no established protocols for the extraction, digestion and quantification of RBC membrane proteins by liquid chromatography based mass spectrometry (LC–MS). The major areas of differentiation in the sequence of steps required for comprehensive RBC ghost preparation for LC MS are in choices related to the use or not of detergents [9–11] membrane isolation procedures including the use of leucocyte filters [12] and sample collection and storage conditions most notably the impact of using RBC preservative fluids [13].

The anuclear erythrocyte is unique amongst all human cell types in that its diversity is primarily determined by its membrane and associated components. Red cell membranes (RCM) are composed of mainly protein and lipid based structures impacting their transport, antigen profiles and mechanical characteristics, all of which can be altered in disease. These mechanical functions involve oxygen transport, adhesion interactions with other cells as well as receptor signalling processes which have been implicated in infection and inflammatory diseases [14]. Red cells constitute the core oxygen transport molecules in humans and are generated via a process of erythropoiesis. This is divided into four stages in a process triggered by low oxygen tension in cells which in turn activate the hormone erythropoietin in a negative feedback loop [15]. Myeloid progenitor cells in the bone marrow become locked into an erythropoietic fate and are released into the blood as reticulocytes which mature into fully fledged erythrocytes staying in circulation for 90–120 days in healthy individuals [16]. Erythrocyte survival and morphology can be severely altered in a number of diseases making them an important target for study [17, 18].

Ghost RCM’s constitute a collection of erythrocytes devoid of their cytosolic content with the retainment of their membrane and cytoskeletal structures. LC–MS based proteomics offers sensitive methods with the capacity to identify a significant proportion of integral and associated membrane proteins. An overview of current literature shows maximal identifications of several hundred proteins at best using described methods [4, 8, 19–21]. A recent publication has proposed combined identifications of over 2500 membrane proteins using an amalgamation of fractionation approaches combined with three sets of multi-enzyme digests [7]. Though insightful, this latest method proposed is labour intensive and unlikely to be applicable for mid-to large-scale clinical discovery cohorts. In this paper, we assess the impact of varying storage conditions on erythrocyte proteomic expression as well as the stability of major membrane proteins and propose a low intervention, high recovery methodology for the comprehensive isolation and characterization of the protein content of erythrocyte membranes. Few investigations have been made into the impact of prolonged storage on red blood cells with the only significant paper using LCMS published choosing to focus on morphological changes and their resultant impact on the quantity and composition of phospholipids [22]. The fundamental approach of the proposed method is in its preference for the limited usage of chemical modulators such as detergents in the purification and recovery of RBC ghosts from whole blood. Despite this, a demonstrated capacity to reproducibly cover a broad spectrum of known membrane and membrane associated proteins in human whole blood is achieved.

## Methods

### Sample collection: stability studies

**Fig. 1:**
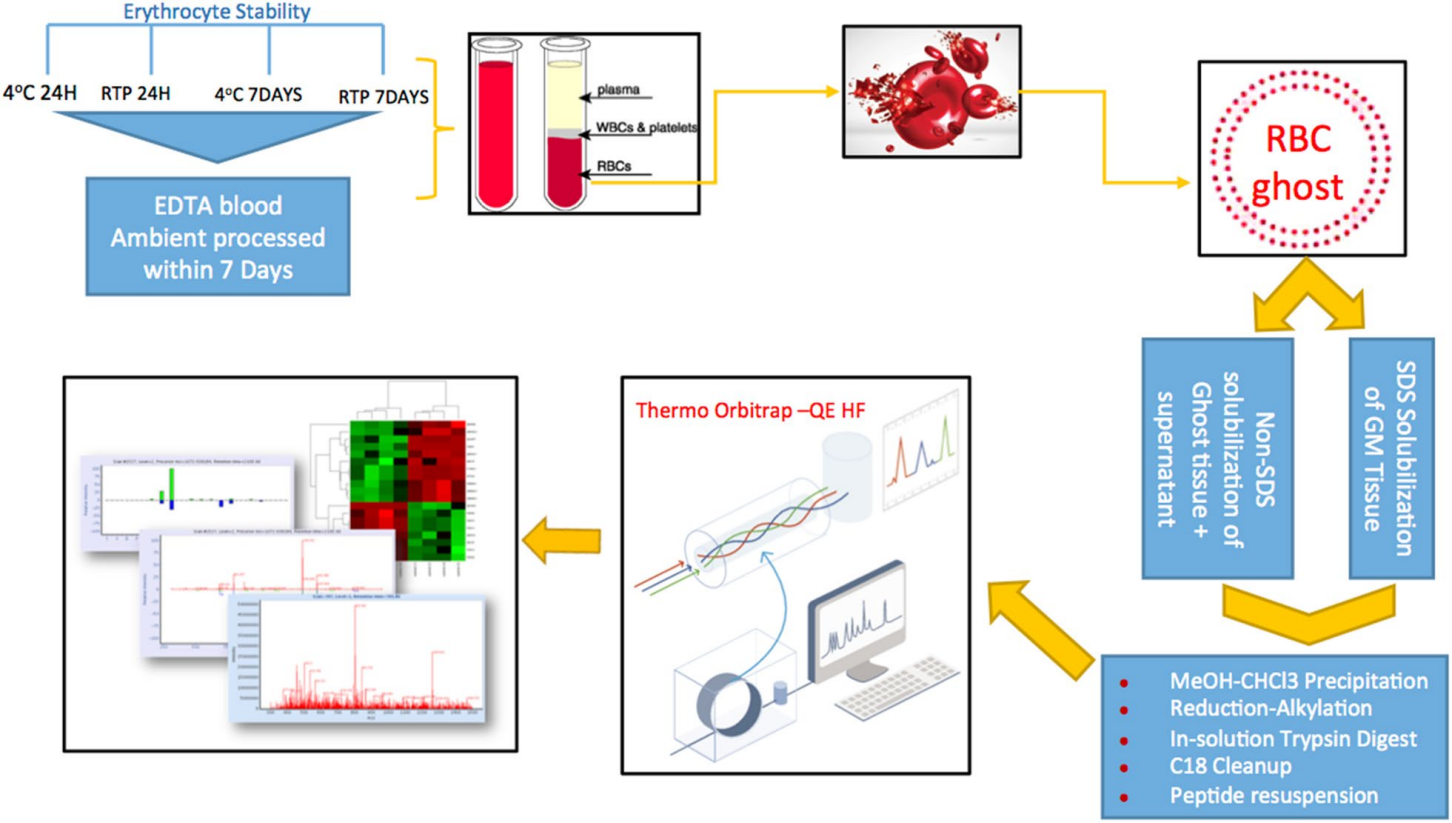
Summary of experimental process

Stability studies were conducted on whole blood collected from three volunteer anonymized healthy donors who gave informed consent. Four vials of 10 millilitres of whole blood were collected into K2EDTA vacutainers via venepuncture. The following storage conditions were assessed on each sample: (1) whole blood kept at 4 °C with packed erythrocyte membranes isolated within 24 h of venepuncture; (2) whole blood kept at room temperature with packed erythrocyte membranes isolated within 24 h of venepuncture (3) whole blood kept at 4 °C for 7 days with packed erythrocyte membranes isolated on day 8 and; (4) whole blood kept at room temperature for 7 days with membrane isolation on day 8 (Fig. 1).

### Sample collection: RBC proteome coverage optimization

Twelve samples were selected from the wider Proteomics Pathways of Sickle Cell Anaemia (P2SCA) study cohort. The study involved the recruitment of one-hundred and twenty one individuals identified from the Muhimbili Sickle Cell Programme database which has since 2004 surveilled Sickle Cell Anaemia (SCA) cases and controls from outpatient clinics and admissions at the Muhimbili National Hospital in Dar es Salaam, Tanzania. Potential participants were approached and informed of the study objectives and those willing to participate gave informed consent. Whole blood was collected into 10 ml K2EDTA vacutainers via venepuncture and the samples shipped within 72 h to the University of Oxford at ambient temperature. Samples were received, logged and processed to isolate plasma, erythrocyte and buffy coat fractions within 7 days of collection. The P2SCA study was granted full ethical clearance by the review boards at the Muhimbili University of Allied Sciences (MUHAS) and the Tanzania Nation Institute for Medical Research (NIMR).

### Erythrocyte ‘ghost’ membrane isolation

Ten millilitres of whole blood was spun for 10 min at 3000 RPM (1731G) to separate packed red cells, plasma and buffy coat. The latter two fractions were removed via aspiration and frozen at − 80 °C, leaving behind the packed erythrocytes which were taken forth for extensive washing and lysis steps to isolate ghost erythrocyte membrane cells.

Approximately 2–4.5 ml of packed erythrocytes were transferred to a 50 ml falcon tube and washed in tenfold volume of 1X PBS. The suspension was mixed by hand inversion and spun at 3000 RPM at 4 °C thrice. Erythrocytes were pelleted after each centrifugation step and the resultant supernatant aspirated. Washed cells from this point were kept on ice for the remaining steps. A lysis buffer of 5 mM Na_2_HPO_4_ at pH 7.4 was prepared and kept at 4 °C before use. The washed RBC pellets were transferred to 50 ml ultracentrifuge tubes; 30 ml of cold lysis buffer were added to each erythrocyte pellet, mixed by strong inversion and spun on a Beckman Coulter ultracentrifuge at 14,000 RPM at 4 °C for 10 min with slow deceleration to avoid ghost pellet disruption. The resulting supernatant was removed and the steps repeated for a total of five washes. 0.5 mM of the protease inhibitor phenylmethylsulfonyl fluoride (PMSF) was added to the lysis buffer at the last wash cycle. The resultant ghost membranes were promptly collected and frozen at − 80 °C.

### Ghost membrane preparations for stability studies

Ghost membranes were resuspended 3:1 in 4× Laemmli Buffer (200 mM Tris–HCl, pH 6.8, 400 mM DTT, 8% SDS, 40% Glycerol and 0.4% Bromophenol Blue) and a Pierce BCA assay kit used to determine protein concentration. Using 80 μg of starting protein amount, the resuspended ghosts underwent reduction-alkylation, double methanol-chloroform precipitation to remove excess detergent and overnight digest with trypsin [23]. Peptide desalting was performed on a Waters C18 solid phase extraction column. Briefly, the column was equilibrated with organic buffer (65% ACN; 35% H_2_O, 0.1% TFA) followed by addition into aqueous buffer (65% H_2_O; 35% ACN, 0.1% TFA) of the peptide digest sample. The bound peptides were then washed with the aqueous buffer and eluted in 1200 μl of organic solution. The eluate was dried down in a vacuum centrifuge and resuspended in aqueous buffer to a final concentration of 2 μg/μl.

### Ghost membrane preparations for RBC proteome optimization and analysis

Ghost membrane suspensions from sample P2S1 were spun down and 1:1 volumes of the semi solid pellet (tissue) and its’ supernatant combined as starting material for the reduction-alkylation, methanol chloroform precipitation and overnight in-solution digestion with trypsin steps. The resultant peptides were quantified using a Thermo kit (Pierce™ Quantitative Colorimetric Peptide Assay) and 50ug from each patient sample loaded on a C18 based SOLA Hydrophobic Reversed Phase (HRP) column. Briefly, the column was equilibrated with organic buffer (65% ACN; 35% H_2_O, 0.1% TFA) followed by addition into aqueous buffer (65% H_2_O; 35% ACN, 0.1% TFA) of the peptide digest sample. The bound peptides were then washed with the aqueous buffer and eluted in 600 μl of organic solution. The eluate was dried down in a vacuum centrifuge and resuspended in aqueous buffer to a final concentration of 2 μg/μl.

### Protein and peptide quantitation assays

Pierce BCA Protein and Quantitative Colorimetric Peptide Assay Kits (Thermo Scientific) were respectively utilized to quantify the protein and peptide concentrations of sample aliquots. Standard kit protocols were followed for each assay with each sample run in duplicate and read on a TECAN microplate reader. The protein BCA assays were read at a wavelength of 570 nm after a 30 min plate incubation at 37 °C. The colorimetric peptide assays were read at 480 nm after a 15 min incubation at 37 °C. Raw data readouts were blank subtracted and the unknown sample absorbances extrapolated from a four parameter non-linear regression quadratic curve fit.

### Mass spectrometry analysis and protein identification

Ultra-performance liquid chromatography (UPLC) separation was performed on a Dionex Ultimate 3000 coupled to a Thermo Scientific Q-Exactive High Field Mass Spectrometer for tandem MS–MS analysis. The UPLC was fitted with an EASY-Spray PepMap C18 column (500 mm × 75 μm, 2 μm particle size) over a 60 min gradient of 2–35% acetonitrile in 5% DMSO, 0.1% formic acid at a flow rate of 250 nl/min. MS1 scans were acquired at a resolution of 60,000 at 200 m/z with the top 12 most abundant precursors targeted for HCD fragmentation. Raw files from each injection were converted to a MASCOT Generic Format (MGF) and searched on the MASCOT server (version 2.5.1) against the UPR Homosapiens database (dated 2017/05/10). The search parameters allowed for 1 missed cleavage, a peptide mass tolerance of ± 10 ppm with carbamidomethyl as a fixed modification on cysteine and the following variable modifications; deamidation (N), deamidation (Q) and oxidation (M). Fragment mass tolerance was set to ± 0.05 Da.

Further analysis of MS raw data was performed using the MaxQuant (v1.6.0.1) [24] and Perseus (v1.6.0.2) [25] programs. Proteins were identified by searching raw data against the UniProt Reference human proteome database (May 2017 version, 93591 sequences) with carbamidomethylation of cysteine as a fixed modification. The variable modifications allowed were oxidation of methionine, and deamidation of asparagine and glutamine. Cleavage specificity was tryptic, allowing for a maximum of two missed cleavages. Enabling the “match between runs” option allowed for identification transfer between samples. Label-free protein quantitation was performed with a minimum ratio count of 2. All other settings used default parameters, which include a peptide and protein false discovery (FDR) rate cut-off of 1%.

The protein groups text files from MaxQuant were used for onward quantitative protein expression analysis across experimental groups. Those not meeting the FDR threshold or identified from the reverse database or contaminant hits were removed prior to further analysis. The resultant list was analysed as per published instructions by the software developers [25]. Statistical analysis on log transformed data were also performed on Perseus.

## Results

### Effects of whole blood storage temperature on erythrocyte membrane protein expression

**Fig. 2:**
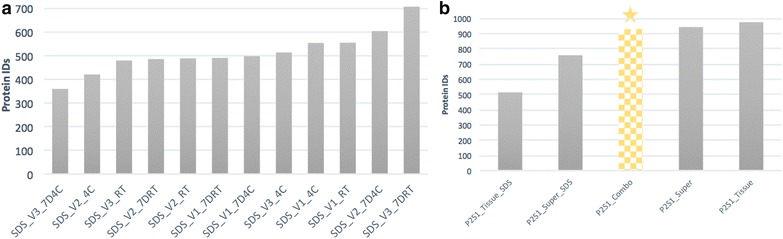
Bar plot showing the total protein numbers identified from MASCOT searches of raw data obtained from: **a** Protein extractions from RBC membranes kept for up to 7 days in different storage conditions; and **b** comparative detergent and non-detergent based RBC membrane extraction methods applied to membrane supernatant “Super”, semi-solid tissue pellets referred to as “Tissue” and a combination of both in samples from the P2SCA cohort. P2S1 acronym stands for Proteomics Pathways Sample #1

**Fig. 3:**
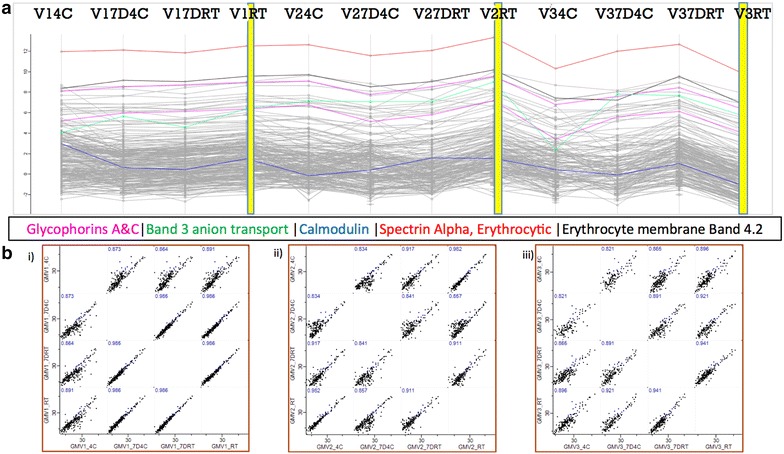
**a** Profile plot showing trends in the quantitative expression levels of major membrane proteins across distinct pre-processing conditions. Lines labelled as follows V (Volunteer) # (participant number) 4C (storage at 4 °C for 24 h); V# 7D4C (storage at 4 °C for 7 days); V# 7DRT (storage at room temperature for 7 days); V# RT (storage at room temperature for 24 h). **b** i–iii Pearson correlation plots of comparative conditions within individual donors

Comparative analysis of the number of protein hits obtained from erythrocyte ghost membrane samples isolated from three volunteers show no significant loss in total proteins identified in whole blood samples stored for up to 7 days (Fig. 2). On a global scale, total protein expression remains relatively stable in whole blood collected and stored for up to 7 days. The results obtained from the stability experiments suggest ambient storage to be more robust for RBC proteomics in comparison to refrigeration. Targeted analysis of the major membrane proteins of different classes are highlighted in Fig. 3a showing that they demonstrate relative stability across conditions. Correlation curves show strong linearity between conditions, as displayed in Fig. 3b i–iii.

### Assessing the effectiveness of utilizing detergent and non-detergent based buffers for membrane solubilization

**Fig. 4:**
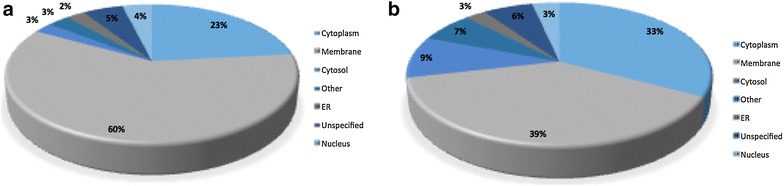
**a** Pie chart summarizing cellular localization of proteins identified from SDS assisted solubilized method. **b** Pie chart summarizing cellular localization of proteins identified from non-SDS assisted solubilized method

**Fig. 5:**
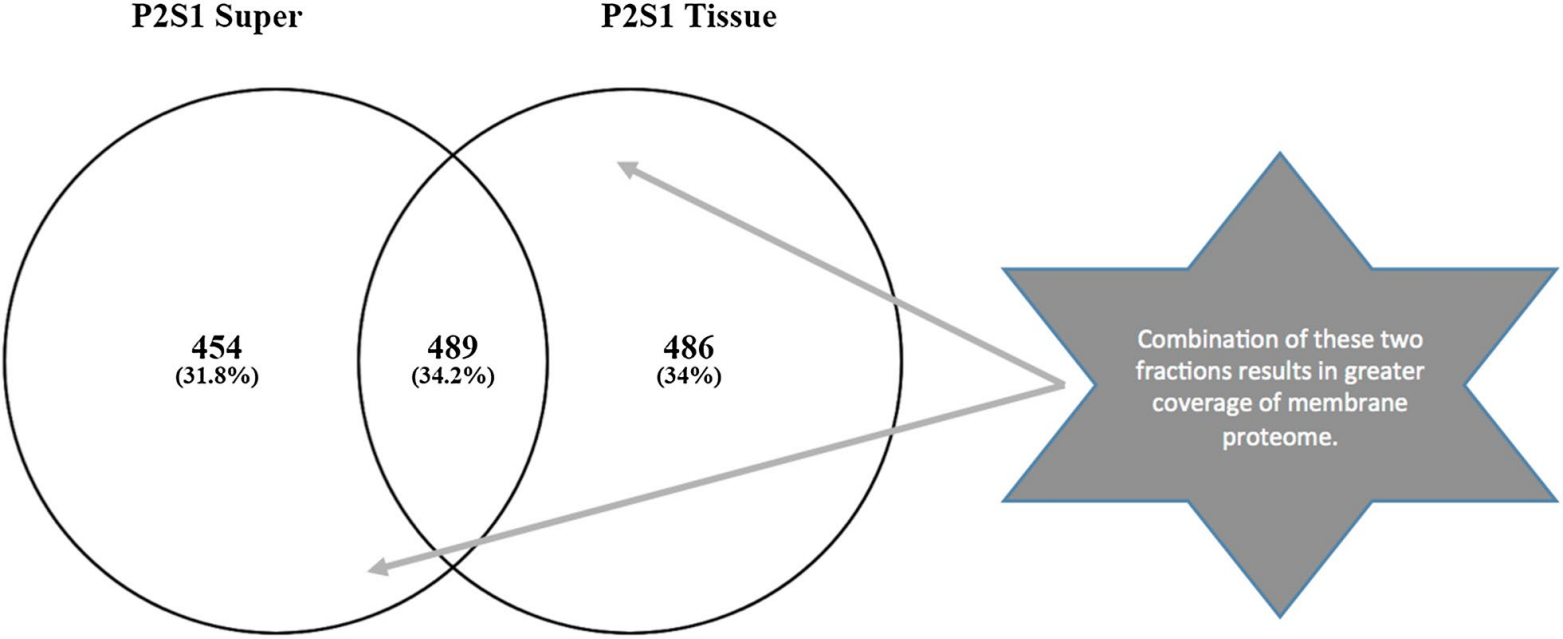
Diagram showing the degree of overlapping proteins expressed in the non-detergent solubilized supernatant and membrane tissue fragments collected following hypotonic lysis assisted ghost membrane preparation of sample P2S1. ‘Super’ refers to the supernatant isolated from isolated ghost membrane cells following centrifugation. ‘Tissue’ refers to the semi-solid pellet collected after this step

The second assessment made in these experiments were the optimal methods to be adopted for extensive coverage of the membrane proteome using detergent and non-detergent assisted solubilization (Fig. 4). A sizeable increase in the number of identifiable proteins was seen by adapting protocols from resuspending ghost pellets in SDS laced buffer to utilizing direct combinations of the semi solid membrane pellets combined with their soluble supernatants (Fig. 2b). The latter approach was tested by assessing the adaptation of tissue versus supernatant preparations. The results obtained show some diversity in the scope of proteins captured (Fig. 5) resulting in the adaptation of a protocol combining both fractions.

### Assessment of membrane proteome coverage and the impact of possible haemoglobin contamination

Manual analysis of the localization of the total range of proteins identified from the optimization experiments show a majority of them categorized as “membrane” or “membrane associated”. The other major proportion were the cytoplasmic fractions, constituting 23% in the SDS-assisted and 33% in the non-SDS assisted method. One of the major concerns in the MS analysis of erythrocyte ghosts is potential contamination with haemoglobin which constitutes > 98% of RBCs. The results obtained from the proposed protocol suggest that the comprehensive washing and lysis steps described above sufficiently lower haemoglobin concentrations thus allowing the quantifiable expression of the diverse erythrocyte proteome. Known membrane proteins such as glycophorins, spectrin (alpha & beta), ankyrin, calmodulin, band 3 anion, glyceraldehyde-3-phosphate dehydrogenase, actin and beta-adducin were the predominant hits obtained in all MASCOT searches with haemoglobin featuring lower down with lower scores. Our method also consistently identified low-abundant membrane proteins such as tropomodulin, several isoforms of the solute carrier family proteins SLC40, SLC43, SLC2A1, ADP/ATP translocase, transmembrane emp24 domain containing protein and calpastatin.

### Robustness of proposed method across serial measurements of proteins from clinical blood samples

Ten individual clinical samples from the P2SCA cohort were searched on the MASCOT server and the sequence coverage of six key membrane proteins tracked as a measure of the robustness of the utilized preparatory and LC–MS analysis methods. Table 1 shows comparable sequence coverage rates across ten independently prepared and analysed patient samples. Analysis of variation (ANOVA) tests on iBAQ quantitative values obtained on Perseus showed no significant difference across protein expression levels of ten independent samples of the same Hb genotype (data not shown). From a starting set of a total 2081 proteins identified across ten samples; several key filtration steps as described above were applied. A rule was set which only allowed proteins identified in 80% of the samples to be included in the final statistical analysis; this yielded a final list of 1296 proteins for comparison. These were normally distributed across the board (Additional file 1: Figure 1).

**Table 1:** Summary of attained sequence coverage across ten independent samples prepared with the finalized detergent free method demonstrating robust identification of major membrane proteins

Accession	Name	Function	Sequence coverage (%) samples 1–10									
			1	2	3	4	5	6	7	8	9	10
P02549	Spectrin alpha chain	RBC membrane skeleton	95	88	88	92	89	90	90	91	93	93
P11277	Spectrin beta chain	RBC membrane skeleton	90	89	90	89	88	91	90	94	90	95
P16157	Human Ankyrin	Integral membrane protein	95	90	93	94	90	91	90	94	95	93
P11171	Protein 4.1	RBC membrane skeleton	77	89	73	71	72	71	75	78	69	76
P02730	Band 3 anion transport	Ion exchange	53	59	67	58	77	69	67	52	67	53
P04406	Glyceraldehyde-3-phosphate dehydrogenase	Glycolytic enzyme	98	84	94	95	94	94	94	93	95	97

## Discussion

Human erythrocytes represent an important cell type relevant to several disease models. Disease and infection associated morbidity has been known to impact the lifespan and morphology of these cells. In their role as oxygen carriers, healthy RBC’s undergo routine cycles of deformation and reformation allowing them to pass through capillaries much smaller in diameter. RBC’s are anuclear cells with their cytosols primarily composed of haemoglobin, this renders their membranes as their major site of differentiation reflecting changes effected by genotype and environmental differences. That said, there exist few methodologies which allow medium to high throughput capacity study of the membrane structures of these cells in a clinical context. The methods proposed here aim to make a contribution towards this shortfall by allowing researchers to undertake these experiments in a reproducible manner.

The high throughput method proposed for comprehensive proteomic analysis of the RBC membrane proteome is novel standing out as a research publication which has assessed the implications of storage duration and condition on the recovery of proteins from erythrocyte ghosts. In addition, with the demonstrable impact of this an optimal protocol with minimal sample interference is also presented allowing for the robust selection of a broad spectrum of membrane-associated proteins without fractionation. The potential impact of the presentation of this simplified and streamlined approach for ghost membrane analysis cannot be overstated and should act as an important step in allowing the analysis of this key fraction of whole blood in numerous disease contexts,

## Conclusions

The results presented demonstrate the stability of the erythrocyte proteome over a defined period; a point which should facilitate sample collection and transport across a range of clinical studies. In the area of sample preparation, the protocol proposed offers low intervention methods compatible with established protein isolation techniques applicable to large sample numbers. Without the use of fractionation and multi-enzyme digestion methods, a large number of robustly identifiable proteins are still reliably covered and quantifiable using standard proteomics analysis packages and tools.

## Additional file

**Additional file 1: Figure** **1** Histogram showing the distribution of the Intensity Based Absolute Quantification (iBAQ) values for identified peptides across samples 1 to 10 (represented by iBAQ 1-10 respectively) listed in Table 1.

## Abbreviations

ANOVA: analysis of variation
FDR: false discovery rate
HRP: hydrophobic reversed phase
LC–MS: liquid chromatography mass spectrometry
MALDI-TOF: Matrix Associated Laser Desorption Ionization Time-of-Flight
MUHAS: Muhimbili University of Allied Sciences
NIMR: National Institute for Medical Research
PMSF: phenylmethylsulfonyl fluoride
P2SCA: Proteomics Pathways of Sickle Cell Anaemia
RBC: red blood cell
RCM: red cell membrane
SCA: sickle cell anaemia
UPLC: ultra-performance liquid chromatography
