# Supplementary figures and images for: A robust mass spectrometry method for rapid profiling of erythrocyte ghost membrane proteomes
Source: Clin Proteomics. 2018 Mar 21;15:14. doi: 10.1186/s12014-018-9190-4 (PMC5863380; doi:10.1186/s12014-018-9190-4)

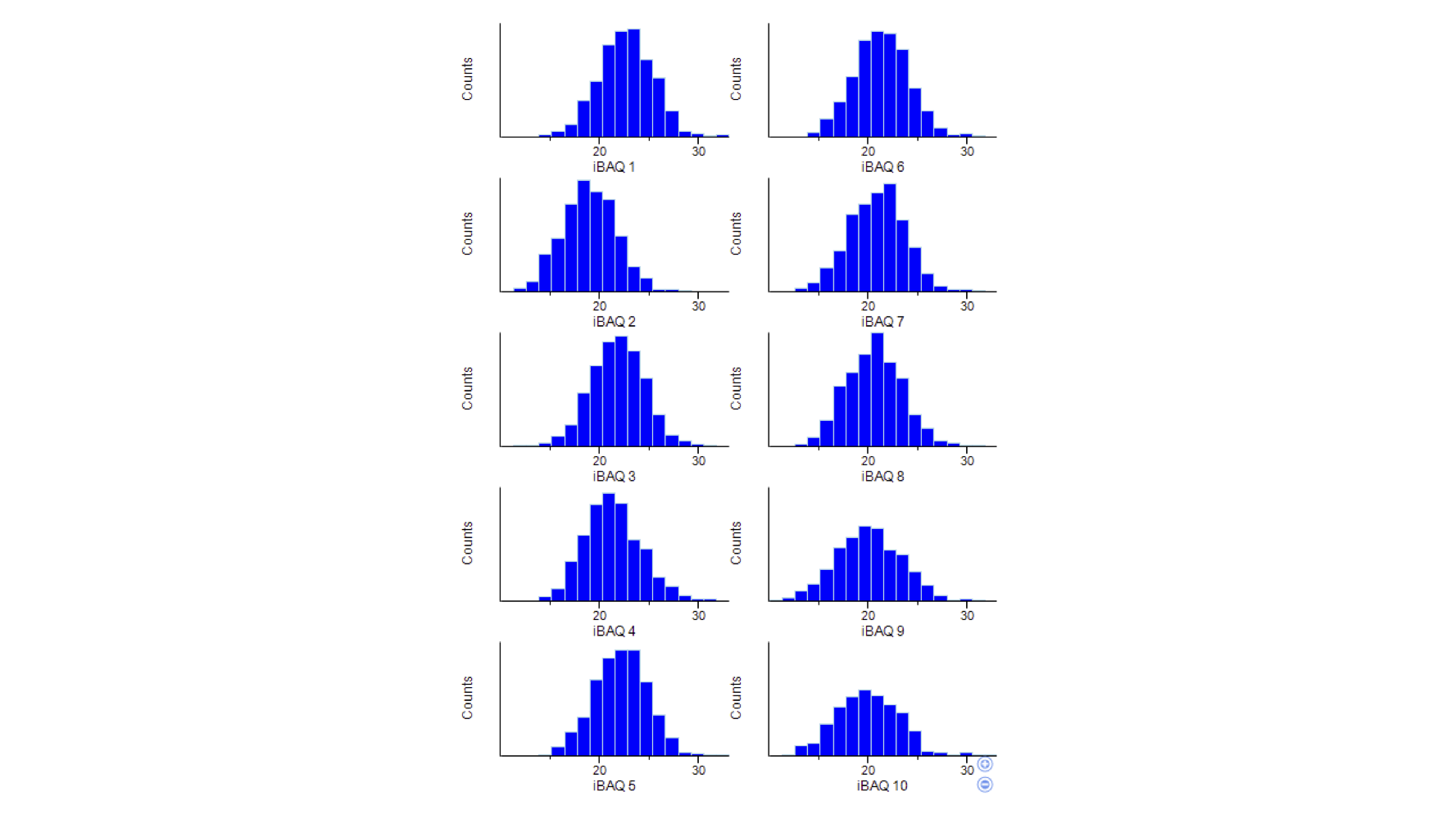

Supplement: Supplementary file 1 — Additional file 1: Figure 1 Histogram showing the distribution of the Intensity Based Absolute Quantification (iBAQ) values for identified peptides across samples 1 to 10 (represented by iBAQ 1-10 respectively) listed in Table 1. [file 12014_2018_9190_MOESM1_ESM.tiff]
